# Organic NMR crystallography: enabling progress for applications to pharmaceuticals and plant cell walls†

**DOI:** 10.1039/d4fd00088a

**Published:** 2024-05-20

**Authors:** Zainab Rehman, Jairah Lubay, W. Trent Franks, Albert P. Bartók, Emily K. Corlett, Bao Nguyen, Garry Scrivens, Brian M. Samas, Heather Frericks-Schmidt, Steven P. Brown

**Affiliations:** a Department of Physics, University of Warwick Coventry CV4 7AL UK S.P.Brown@warwick.ac.uk; b Pfizer Worldwide R&D Sandwich Kent UK; c Pfizer Worldwide R&D Groton CT USA; d Warwick Centre for Predictive Modelling, School of Engineering, University of Warwick Coventry CV4 7AL UK

## Abstract

The application of NMR crystallography to organic molecules is exemplified by two case studies. For the tosylate salt of the active pharmaceutical ingredient, Ritlectinib, solid-state NMR spectra are presented at a ^1^H Larmor frequency of 1 GHz and a magic-angle spinning (MAS) frequency of 60 kHz. Specifically, ^14^N–^1^H heteronuclear multiple-quantum coherence (HMQC) and ^1^H–^1^H double-quantum (DQ) single-quantum (SQ) correlation experiments are powerful probes of hydrogen bonding interactions. A full assignment of the ^1^H, ^13^C and ^14^N/^15^N chemical shifts is achieved using also ^1^H–^13^C cross polarization (CP) HETCOR spectra together with gauge-including projector augmented wave (GIPAW) DFT calculation for the geometry-optimised X-ray diffraction crystal structure that is reported here (CCDC 2352028). In addition, GIPAW calculations are presented for the ^13^C chemical shifts in the two polymorphs of cellulose for which diffraction structures are available. For both case studies, a focus is on the discrepancy between experiment and GIPAW calculation.

## Abstract 2

The application of NMR crystallography to organic molecules is exemplified by case studies for a pharmaceutical salt and cellulose polymorphs.

## Graphical abstract

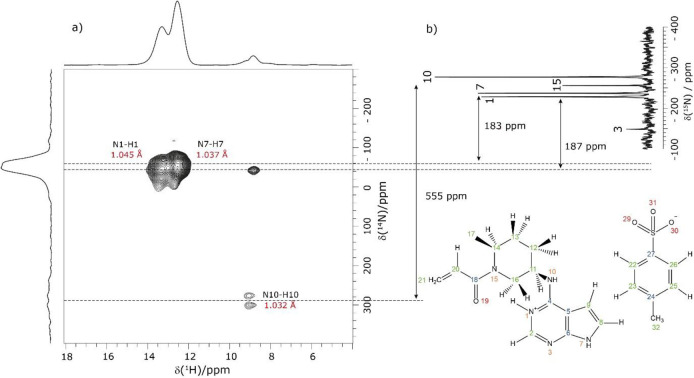

## Introduction

Built upon the DFT gauge-including projector augmented wave (GIPAW) method,^1–3^ the value of NMR crystallography for analysis of solid-state structures of organic molecules is being increasingly recognised. This paper aims to take stock of where the field is today, notably considering that experimental solid-state NMR can now readily access magnetic fields corresponding to a ^1^H Larmor frequency of at least 1 GHz and magic-angle spinning (MAS) frequencies of at least 60 kHz. The paper identifies current challenges and points to new approaches under consideration. The focus is on applications to pharmaceuticals,^4^ but suitability for aiding in the interpretation of solid-state NMR spectra of plant cell walls^5^ is also considered.

For the calculation of chemical shieldings for the spin *I* = 1/2 nuclei ^1^H and ^13^C, there is an extensive literature that the collaborative computational project for NMR crystallography (CCP-NC) database^6^ based on the .magres format^7^ is endeavouring to bring into one place. From this extensive literature, it is well established that the discrepancy with respect to experiment is usually within 1% of the chemical shift range, *i.e.*, within ∼0.2 ppm and ∼2 ppm for ^1^H and ^13^C chemical shifts, respectively.^3,8,9^

That said, there are challenges. It is known that the gradient of a plot of experimental isotropic chemical shift against GIPAW calculated absolute shielding deviates from minus one,^10^ and there is disagreement in the community as to how referencing should be carried out. It is to be noted that this referencing problem is circumvented by a recently published method that considers differences in calculated chemical shielding between solution and the solid state. Such a difference does not require referencing, and an evaluation of correlation with respect to the corresponding change in experimental chemical shift between solution and solid enables the differentiation of solid-state form.^11,12^ We also note that larger discrepancies between experiment and GIPAW calculation have been systematically observed for specific chemical groups, notably for OH⋯O ^1^H and N=C–N ^13^C chemical shifts.^13^ Moreover, there remains the challenge that GIPAW calculation at an effective temperature of 0 K does not reproduce the known temperature dependence of hydrogen-bonded ^1^H chemical shifts.^14–17^ An important quadrupolar (*I* ≥ 1) nucleus for studying hydrogen bonding interactions in organic solids is ^14^N for which ^1^H detection is important;^18–22^ DFT calculation is valuable for prediction of the electric field gradients that determines the quadrupolar parameters that affect the solid-state NMR spectra.

## A review of applications of NMR crystallography to pharmaceutical molecules

As one of the fathers of the field of NMR crystallography, alongside Francis Taulelle,^23^ Robin Harris focused on applications to small and moderately sized organic molecules, notably, pharmaceuticals.^24,25^ Early applications of the GIPAW method were, with Chris Pickard, Francesco Mauri and Jonathan Yates, for the calculation of ^1^H, ^13^C and ^19^F chemical shifts in the pharmaceutical flurbiprofen, presented with MAS NMR spectra,^26^ and, together with Lyndon Emsley, for the calculation of ^13^C chemical shifts for testosterone for the two distinct molecules in the asymmetric unit cell, presented with two-dimensional ^13^C refocused INADEQUATE MAS NMR spectra.^27^

Applications to pharmaceuticals up to 2018 are referred to in the comprehensive review of NMR crystallography of organic solids by Hodgkinson;^9^ here, we refer to some specific highlights. The added value of an NMR crystallography approach for quantifying intermolecular interactions, notably hydrogen bonding, was demonstrated by calculations of the change in chemical shift between a GIPAW calculation for the full crystal structure and an isolated molecule for phenylphosponic acid by Gervais *et al.*,^28^ for maltose anomers by Yates *et al.*,^29^ and by Bradley *et al.* for the pharmaceutical indomethacin.^30^ A significant advance was the coupling of NMR crystallography with crystal structure prediction (CSP) by Emsley and Day and co-workers, whereby, as demonstrated for thymol, best agreement to the putative CSP structures was obtained *via* determining the root mean squared error (RMSE) between experimental and GIPAW calculated ^1^H and ^13^C chemical shifts.^31^ The importance of NMR crystallography to the pharmaceutical industry is demonstrated by a growing number of publications in collaboration with scientists from pharmaceutical companies, for example to sibenadet polymorphs with AstraZeneca^32^ and to cimetidine and tenoxicam with GlaxoSmithKline,^33^ both in 2012. The potential to incorporate dispersion correction into DFT calculations in the DFT-D approach was demonstrated by Dudenko *et al.* for indomethacin in 2013.^34^ As an alternative to the GIPAW planewave method, Beran and co-workers have advocated for a fragment-based approach that permits the use of hybrid functionals such as PBE0.^35–38^ A major advance whose significance is ever increasing was the development in 2018 by Ceriotti, Emsley and co-workers of the Shift-ML method for predicting chemical shifts by applying machine learning based on a training set of GIPAW calculated chemical shifts.^39^

Focusing on the last five years since 2019, there have been a range of impressive applications of NMR crystallography and methodological advances. Combining NMR crystallography, including two-dimensional ^1^H–^13^C and ^1^H–^15^N HETCOR MAS NMR spectra, with electron diffraction, Guzman-Afonso *et al.* have identified the hydrogen bonding network in form B of the pharmaceutical cimetidine.^40^ Bartova *et al.* have combined calculation with experiment, notably ^14^N–^1^H two-dimensional MAS NMR spectra, to study tautomerism in azo dyes, focusing on hydrogen bonding interactions.^41^ Scarperi *et al.* have used NMR crystallography to study the pharmaceutical carbimazole, presenting ^1^H double-quantum (DQ) and ^1^H–^13^C heteronuclear correlation MAS NMR spectra.^42^ Dudek *et al.* have used NMR crystallography with ^1^H DQ MAS NMR spectra to probe the co-crystal landscape when an AB binary system (barbituric acid : thiobarbituric acid) is perturbed by a crystalline synthon C (1-hydroxy-4,5-dimethyl-imidazole 3-oxide) in a ball mill.^43^ Dudek and co-workers and Pawlak *et al.* have also combined NMR crystallography with CSP for co-crystals of the antibiotic linezolid^44^ and for the pharmaceutical teriflunomide.^45^ Mathew *et al.* have presented an NMR crystallography study of the pharmaceutical sitagliptin phosphate monohydrate including ^13^C–^13^C and ^13^C–^15^N MAS NMR correlation spectra recorded at natural abundance using dynamic nuclear polarisation.^46^ Brouwer and Mikolajewski have recently presented GIPAW calculations along with ^1^H DQ and ^1^H–^13^C heteronuclear correlation MAS NMR spectra for glucose, to identify trends in the ^1^H chemical shift with hydrogen bonding parameters,^47^ noting that Shen *et al.* have presented GIPAW calculations to complement ^17^O MAS NMR experiments for the same sugar molecule.^48^ Chierotti and co-workers have combined experiment such as ^1^H DQ and ^1^H–^13^C heteronuclear correlation as well as ^14^N–^1^H MAS NMR spectra, and GIPAW calculation to study co-crystals of the pharmaceutical ethionamide,^49^ probe tautomerism in the pharmaceutical mebanazole,^50^ identify zwitterions, in combination with CSP, in isomers of pyridine dicarboxylic acid,^51^ and to analyse leucopterin, the white pigment in butterfly wings, including a ^1^H DQ MAS spectrum at 1 GHz.^52^ Working together with scientists at AstraZeneca and Pfizer, Brown and co-workers have presented NMR crystallography studies of a range of pharmaceutical molecules.^4,33,53–56^

Together with Dracinsky, Hodgkinson has advocated for bringing together of molecular dynamics and nuclear quantum effects in the path-integral molecular dynamics (PIMD) approach.^57^ This proves important for predicting salt or co-crystal formation corresponding to the transfer or not of a proton, as evidenced by the ^1^H chemical shift.^58,59^ Dracinsky has investigated geometry optimisation and GIPAW NMR calculation using the hybrid functional B3LYP or the meta-GGA functional rSCAN^60^ and observed improved agreement compared to experiment for ^1^H and ^13^C chemical shifts.^61^ This analysis has been extended to NMR crystallography of amino acids.^62^ Recently, building upon the use of a molecular correction term with a hybrid density functional,^63^ Iuliucci *et al.* have compared the agreement compared to experiment for computationally more expensive double hybrid or Moller–Plesset perturbation theory (MP2), with no advantage for the test set of ^13^C and ^15^N chemical shifts being observed.^64^ Schurko and co-workers have investigated how hybrid density functionals can improve agreement with respect to experiment for the ^13^C chemical shielding tensor for the pharmaceutical cimetidine.^65^ Recently Holmes *et al.* have compared the agreement to experiment for the ^13^C chemical shielding tensor for five nitrogen-dense compounds when employing the hybrid functional PBE0 or the double-hybrid functional PBE0-DH.^66^ Emsley and co-workers have published a series of impressive papers that make use of the ShiftML resource. Bayesian statistical theory has been integrated into the use of NMR chemical shifts,^67,68^ and to enhance crystal structure prediction protocols.^69,70^ Chemical-shift dependent interaction maps based on ShiftML have been presented.^71^ Working with scientists at AstraZeneca, structural insight has been derived for amorphous pharmaceuticals.^72,73^

## Experimental and computational details

### Solid-state NMR

Experiments were performed using a Bruker Avance III, a Bruker Avance II+, and a Bruker NEO spectrometer operating at a ^1^H Larmor frequency of 500.0 MHz, 600.0 MHz, and 1000.0 MHz, respectively, corresponding to a ^13^C Larmor frequency of 125.8 MHz, 150.9 MHz, and 251.5 MHz, respectively. ^14^N–^1^H experiments were performed at a ^1^H Larmor frequency of 600 MHz and a ^14^N Larmor frequency of 43.4 MHz. A 1.3 mm HXY probe at 60 kHz MAS and a 4 mm HXY probe at 12.5 kHz MAS, both in double resonance mode, were utilised. The ^1^H 90° pulse duration was 2.5 μs corresponding to a ^1^H nutation frequency of 100 kHz. SPINAL-64 ^1^H heteronuclear decoupling^74^ was employed during the acquisition of a ^13^C or ^15^N FID. In all 2D experiments, States-TPPI was used to obtain sign-discrimination in *F*_1_. A recycle delay of 12 s was used.

^1^H–^13^C 1D Cross-Polarisation (CP) MAS NMR and 2D CP Heteronuclear Correlation (HETCOR) MAS NMR at 600 MHz and 1 GHz. For CP at 12.5 kHz MAS, CP was achieved using a ramp (70–100%).^75^ The nutation frequencies for ^1^H and ^13^C, respectively, during CP were approximately 100 kHz and 80 kHz at 600 MHz and 12.5 kHz MAS and 50 kHz and 10 kHz at 1 GHz and 60 kHz MAS. The SPINAL-64 pulse duration was 5.1 μs at 12.5 kHz MAS and 45.8 μs at 60 kHz MAS. The phase cycling employed was as follows: ^1^H 90° pulse (90° 270°), ^13^C CP contact pulse (2{0°} 2{180°} 2{90°} 2{270°}), receiver (0° 180° 180° 0° 90° 270° 270° 90°).

For HETCOR at 1 GHz and 60 kHz MAS, no homonuclear ^1^H decoupling was applied in *t*_1_. 1 GHz spectra were recorded with low-power ^13^C rf. irradiation during CP at an irradiation frequency of 50 ppm or 120 ppm. Here, 32 transients were co-added for each of the 128 (^13^C at 120 ppm) or 192 (^13^C at 50 ppm) *t*_1_ FIDs using a *t*_1_ increment of 50 μs, resulting in an experimental time of 14 or 21 hours.

^1^H–^15^N 1D Cross-Polarisation (CP) MAS NMR. CP was achieved using a ramp on ^1^H (50–100%),^75^ with the same phase cycling as for the ^1^H–^13^C experiments. The nutation frequencies for ^1^H and ^15^N during CP were 70 kHz and 25 kHz. The SPINAL-64 pulse duration was 5.3 μs at a ^1^H nutation frequency of 100 kHz.

Fast MAS (60 kHz) ^1^H–^1^H 2D NMR Experiments at 600 MHz and 1 GHz. ^1^H–^1^H double quantum (DQ) spectra with one rotor period of BaBa recoupling^76,77^ were acquired using a rotor-synchronised *t*_1_ increment of 16.67 μs. In both cases, 48 transients were co-added for each of the 128 *t*_1_ FIDs, corresponding to an experimental time of 21 hours. A 16-step phase cycle was implemented, with Δ*p* = ±2 selected during DQ excitation (4 steps) and Δ*p* = −1 on the *z*-filter 90° pulse (4 steps), where *p* is the coherence order. The phase cycling employed was as follows: ^1^H BABA pulses (0° 90° 180° 270°), ^1^H 90° (*z*-filter) (4 {0°} 4{90°} 4{180°} 4{270°}), receiver (0° 180° 0° 180° 90° 270° 90° 270° 180° 0° 180° 0° 270° 90° 270° 90°).

2D ^14^N–^1^H heteronuclear multiple-quantum coherence (HMQC)^18–22^ MAS (60 kHz) NMR experiments. These were acquired with 8 rotor periods (133.6 μs), 16 rotor periods (267.2 μs) and 24 rotor periods (400.8 μs) of phase-inverted R^3^ recoupling with +*x* −*x* phase inversion for every rotor period of the *n* = 2 (*ν*_1_ = 2*ν*_R_) rotary resonance recoupling pulses.^19,22,78–81^ The ^14^N pulses were both of duration 11 μs. A rotor-synchronised *t*_1_ increment of 16.67 μs was used. The experiments were obtained with 32 coadded transients for each of the 256 *t*_1_ FIDs, corresponding to 27 hours experimental time. A 4-step nested phase cycle was used to select changes in the coherence order Δ*p* = ±1 on the first ^1^H pulse (2 steps) and Δ*p* = ±1 on the last ^14^N pulse (2 steps).

Referencing. The ^13^C and ^1^H chemical shifts were referenced with respect to tetramethylsilane (TMS) using l-alanine at natural abundance as the secondary reference. The CH_3_ group of l-alanine is referenced at 1.1 ppm for the ^1^H methyl resonance and 177.8 ppm for the ^13^C carboxylate resonance. This corresponds to adamantane at 1.85 ppm for ^1^H^82^ and 38.5 ppm for ^13^C.^83^ The ^14^N shifts were referenced with respect to saturated NH_4_Cl aqueous solution using β-aspartyl-l-alanine at natural abundance, whereby the NH resonance is at −284 ppm at a ^1^H Larmor frequency of 600 MHz, corresponding to liquid CH_3_NO_2_ at 0 ppm.^21,84^ The ^15^N chemical shifts are also referenced to liquid CH_3_NO_2_ at 0 ppm.^85^ For equivalence to the chemical shift scale frequently used in protein ^15^N NMR, where the alternative IUPAC reference (see Appendix 1 of ref. 86) is liquid ammonia at 50 °C, it is necessary to add 379.5 ppm to the given values.^87^ The accuracy of the experimental shifts is within ±0.2, ±0.1 and ±5 for ^1^H, ^13^C and ^15^N, and ^14^N, respectively.

### GIPAW calculations

Density functional theory (DFT) calculations were performed using CASTEP^88^ version 19.1 for 1 and version 20.1 or 22.1 for the cellulose polymorphs. For the full crystal, geometry optimisation with fixed unit cell parameters followed by magnetic shielding calculations to determine the NMR parameters were completed. Distances stated in this paper are for the geometry optimised crystal structure. The Perdew–Burke–Ernzerhof (PBE) exchange correlation functional,^89^ a plane-wave basis set with ultrasoft pseudopotentials and a plane-wave cut-off energy of 800 eV were implemented. The calculations for the cellulose polymorphs were repeated using the regularised version of the SCAN functional,^90^ rSCAN.^60^ A maximum Monkhorst–Pack grid spacing of 2π × 0.1 Å^−1^ or 2π × 0.05 Å^−1^ was used. The GIPAW^1,2^ method was used to calculate the NMR parameters: calculated isotropic chemical shifts were determined from the calculated chemical shieldings according to *δ*_iso_calc_ = *σ*_ref_ − *σ*_calc_. It is noted that it is common practice to calculate a specific reference shielding for each system (see, *e.g.*, Table S8 of ref. 39), though average values over a range of compounds are also available.^38^ For 1, ^13^C, different reference shieldings were used for high- and low-ppm chemical shifts:^91^ 172 ppm for >45 ppm and 175 ppm for <45 ppm. For 2, the reference shieldings for the different calculations are stated in the results Table 5. For ^1^H and ^15^N, a reference shielding of 31 ppm and −160 ppm was used, respectively.

## Case study 1: the active pharmaceutical ingredient Ritlectinib tosylate

**Scheme 1:**
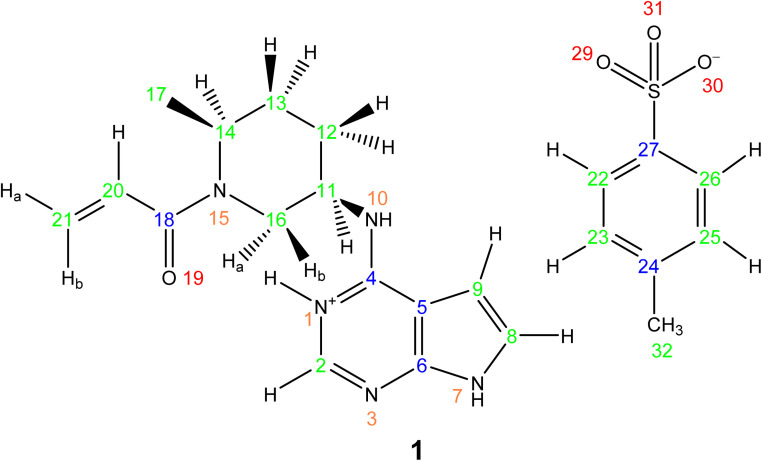


This section showcases current state-of-the-art experimental solid-state NMR for the application of NMR crystallography to moderately sized active pharmaceutical ingredients (APIs). The API, Ritlecitinib,^92^ functions as a selective and irreversible JAK3 inhibitor for irritable bowel disease with additional studies in progress for further uses as a treatment for alopecia areata^93^ and Crohn's disease.^94^ The irreversible binding is covalent in nature to a specific cysteine residue (Cys-909) within the JAK3 protein.^95^ The original synthesis for the molecule, Ritlectinib, was described by Thorarensen *et al.*^96^ In this work, the API is considered in its tosylate salt form, 1 (see Scheme 1).^97^

**Fig. 1:**
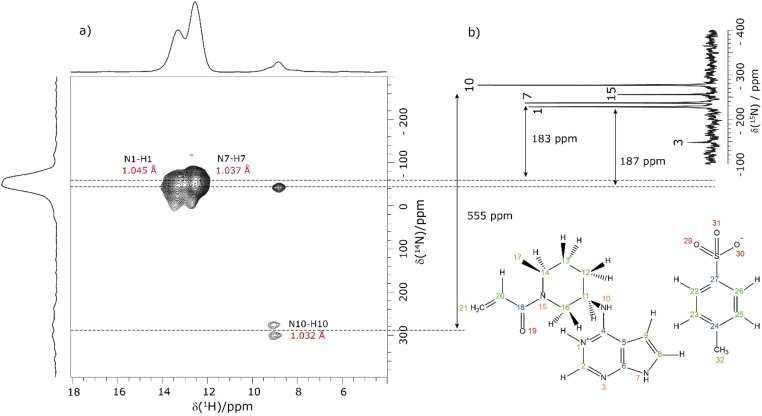
(a) A ^14^N–^1^H (600 MHz) HMQC MAS (60 kHz) NMR spectrum with skyline projections of 1 recorded with 16 rotor periods of phase-inverted R^3^ recoupling, *τ*_RCPL_ = 267.2 μs. (b) Comparison to a 1D ^1^H (500 MHz)–^15^N CP (3.5 ms) MAS (12.5 kHz) NMR spectrum of 1 acquired with 10 240 co-added transients. The arrows indicate the difference between the ^14^N shift and the ^15^N chemical shifts for N1, N7 and N10.

NMR crystallography is particularly well suited to the probing of intermolecular hydrogen bonding that is a key driver of the specific crystal packing adopted in the solid state. Advantage is taken of the marked sensitivity of the ^1^H chemical shift and also the ^14^N/^15^N chemical shift and the ^14^N quadrupolar interaction to hydrogen bonding.^22,33,98,99^ This is illustrated for 1 in Fig. 1 that presents a two-dimensional ^14^N–^1^H HMQC^18–22^ (Fig. 1a) and a ^1^H–^15^N cross polarization (CP) (Fig. 1b) MAS NMR spectrum. Note that there are two NMR-active nuclei for nitrogen, ^14^N and ^15^N, with natural abundances of 99.6% and 0.4%, respectively, whereby the ^15^N nucleus has spin *I* = 1/2, while the ^14^N nucleus has spin *I* = 1. The NMR spectra of nuclei with *I* ≥ 1 are affected by strong quadrupolar interactions between the electric quadrupole moment of the nucleus and the surrounding electric field gradient.

In Fig. 1a, intense ^14^N–^1^H correlation peaks are observed at a ^1^H chemical shift of 12.8 and 13.6 ppm for a ^14^N shift of −45 and −40 ppm, respectively, that are assigned (see below discussion) to the N7–H7 and N1–H1 directly bonded pairs of dipolar-coupled nuclei. As illustrated in Fig. 1 by the double-headed arrows, this corresponds to a change as compared to the ^15^N chemical shifts observed in Fig. 1b of 187 and 183 ppm, respectively. This difference arises because the ^14^N shift is the sum of the isotropic chemical shift (that to a good approximation is the same for ^14^N and ^15^N) and the isotropic second-order quadrupolar shift whose magnitude depends on the strength of the quadrupolar interaction (and is also inversely proportional to the *B*_0_ magnetic field).^21^ The assignment of the observed peaks is made on the basis of a DFT calculation using the GIPAW method as implemented within the CASTEP program. By taking as input a DFT geometry-optimised crystal structure of 1, the GIPAW calculation yields the chemical shielding and the electric field gradient for each nucleus both of which depend on the electronic environment. Table 1 lists the experimental and GIPAW calculated ^14^N and ^15^N NMR parameters for 1. It is observed that the experimental quadrupolar product is very similar for N1 and N7 at 2.6 and 2.5 MHz, respectively, which is ∼20% bigger than the calculated magnitudes of 2.2 and 2.1 MHz, respectively.

**Table 1:** Experimentally determined ^15^N chemical shifts and ^14^N shifts (at a ^14^N Larmor frequency of 43.3 MHz) of 1 from Fig. 1, along with the GIPAW calculated parameters

Atom no.	*δ*(^15^N)_exp_^a^ (ppm)	*δ*(^15^N)_calc_^b^ (ppm)	*δ*(^14^N)_exp_^c^ (ppm)	*δ* ^Q^_iso_(^14^N)_exp_^d^ (ppm)	*P*_Qexp_ e (MHz)	*P*_Qcalc_ f (MHz)
1	−228.3	−227.2	−45	183	2.6	−2.2
3	−148.8	−147.5	—	—	—	−4.0
7	−237.1	−227.3	−40	187	2.5	−2.1
10	−277.1	−278.3	278	555	3.8	−3.8
15	−256.1	−249.3	—	—	—	−4.2

a ^15^N isotropic chemical shift values as taken from the ^1^H–^15^N CP MAS spectrum presented in Fig. 1b.

b *δ*_iso_ = *σ*_ref_ − *σ*_iso_, where *σ*_ref_ = −160 ppm.

c Centre of gravity of the ^14^N peaks extracted from the ^14^N–^1^H HMQC spectrum presented in Fig. 1a. Here, the error is estimated to be within ±5 ppm.

d *δ* ^Q^_iso_(^14^N)_exp_ = *δ*(^14^N)_exp_ − *δ*(^15^N)_exp_.

e *P*_Qexp_ is calculated from *δ*^Q^_iso_(^14^N)_exp_ using the equation: *δ*^Q^_iso_ = (3/40)(*P*_Q_/*v*_0_)^2^ × 10^6^, where 
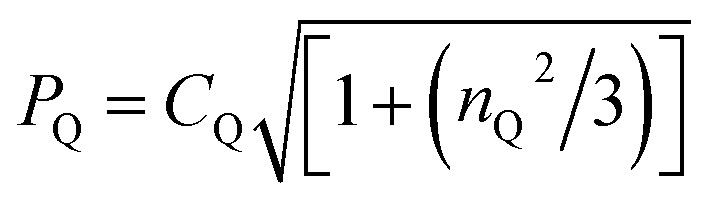
.^19,21,100^ Note that the sign of *P*_Q_ cannot be determined experimentally.

f DFT calculation for the geometry-optimised crystal structure of 1 (CCDC 2352028).

Lower intensity peaks are also observed at a ^1^H chemical shift of 9.2 ppm that corresponds to the H10 atom that is directly bonded to the N10. The peak at a ^14^N shift of −40 ppm corresponds to a longer range N⋯H proximity between N1 and H2 that is bonded to the neighbouring atom in the six-membered aromatic ring. The observation of this correlation peak enables the assignment of the N1–H1 cross peak, that is not possible based on the GIPAW calculation of the nitrogen chemical shift. Note that the calculated values of N1 and N7 are within 0.1 ppm, whereas the experimental ^15^N chemical shifts differ by 8.8 ppm (see Table 1). A low intensity N10–H10 correlation peak is observed at a ^14^N shift of 278 ppm. In this context, note that the quadrupolar coupling constant is significantly bigger for N10 (see Table 1) and that the intensity in such spectra depends on the choice of pulse duration for the two ^14^N pulses, with the optimum value dependent on the quadrupolar coupling constant.^101^ No cross peaks are observed for the N3 and N15 sites for which there is not a directly attached hydrogen atom. Peak intensity in a ^14^N–^1^H HMQC MAS NMR spectrum depends on the recoupling of ^14^N–^1^H dipolar couplings, here using the phase-inverted R^3^ method.^19,22,78–81^ Fig. S3 in the ESI† compares the ^14^N–^1^H HMQC MAS NMR spectrum in Fig. 1a to two other spectra recorded with different durations of R^3^ recoupling of the ^14^N–^1^H dipolar couplings.

Considering the ^1^H–^15^N CP MAS NMR spectrum in Fig. 1b, note that in a CP MAS spectrum, the peak intensity depends on the transfer of transverse magnetisation from ^1^H to ^15^N during the CP contact time. The build-up of CP signal as a function of the contact time depends on the ^1^H–^15^N dipolar couplings that also determine the loss of signal due to *T*_1ρ_ relaxation during the ^1^H spin-lock pulse. Hence different build-up behaviour is observed for the protonated and non-protonated nitrogen resonances, *i.e.*, CP MAS spectra are not quantitative. In Fig. 1b, while the non-protonated N3 and N15 resonances are observed, it is evident that they have lower intensity than that is observed for the protonated N1, N7 and N10 resonances.

**Fig. 2:**
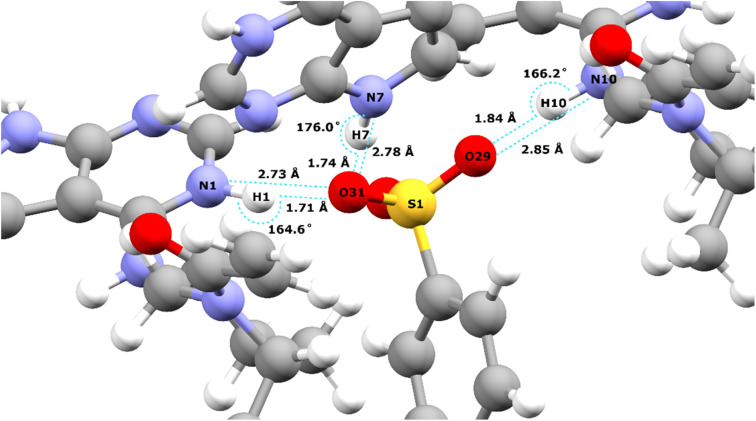
Intermolecular NH⋯O hydrogen bonds in the DFT (CASTEP) geometry optimised crystal structure of 1 (CCDC 2352028) between the oxygen atoms of the tosylate salt and the three NH protons of the API free base (see Table 2 for the hydrogen bond distances and angles).

Table 2 lists the hydrogen bond parameters, namely the N⋯O and H⋯O distances as well as the NHO angles for the three intermolecular NH⋯O hydrogen bonds formed between the three NH moieties and oxygen atoms of the tosylate anion (see also Fig. 2). Note that the H⋯O distances are the similar (1.71 and 1.74 Å) for the N1–H1⋯O31 and the N1–H7⋯O31 hydrogen bonds formed by NH groups on two different API molecules with the same acceptor oxygen atom of one tosylate anion. Table 2 also compares the experimental and GIPAW calculated ^1^H chemical shifts for the three NH groups in 1. The NH GIPAW calculated ^1^H chemical shifts are at least 0.7 ppm higher than the experimental ^1^H chemical shifts. This is a consequence of the well-established temperature dependence of such hydrogen-bonded ^1^H chemical shifts in both solution^102–105^ and solid-state NMR,^14–17^ whereby the ^1^H chemical shift increases upon decreasing temperature, *i.e.*, if the experimental measurement could be performed at close to 0 K, better agreement to the GIPAW calculation that corresponds to 0 K would be expected. In this regard, further note that the GIPAW calculated ^1^H chemical shift is higher for H7 than for H1 (14.7 as compared to 14.3 ppm), while, experimentally, H1 has the higher ^1^H chemical shift, noting the above discussion of the assignment based on the cross peak to N10 observed in Fig. 1a.

**Table 2:** Hydrogen bonding distances and angles from the geometry-optimised crystal structure of 1 (CCDC 2352028, see Fig. 2) and experimental and GIPAW calculated ^1^H NMR chemical shifts for the NH protons

Atom 1	Atom 2	Atom 3	Distance [N⋯O] (Å)	Distance [H⋯O] (Å)	Angle [NHO] (°)	Expt. *δ*(^1^H) (ppm)	Calc. *δ*(^1^H) (ppm)
N10	H10	O29	2.85	1.84	166.2	9.2	9.9
N1	H1	O31	2.73	1.71	164.6	13.6	14.3
N7	H7	O31	2.78	1.74	176.0	12.8	14.7

**Fig. 3:**
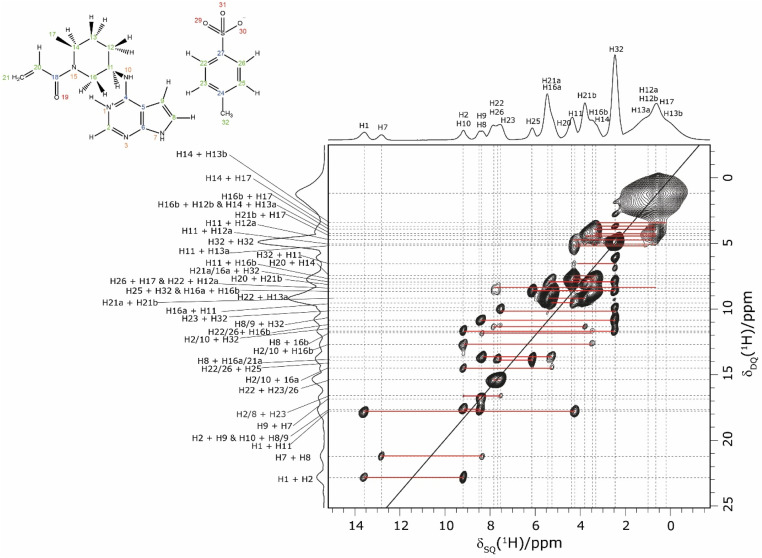
A ^1^H (1 GHz) DQ–SQ 2D MAS (60 kHz) NMR spectrum of 1 with skyline projections recorded with one rotor period of BaBa recoupling. The base contour level is at 4% of the maximum peak height.

In an NMR crystallography study of a pharmaceutical, further insight is obtained by carrying out a ^1^H–^1^H double-quantum (DQ) single-quantum (SQ) homonuclear correlation MAS NMR experiment, as presented for 1 in Fig. 3 that was recorded at a ^1^H Larmor frequency of 1 GHz. The creation of DQ coherence between two ^1^H spins relies on a dipolar coupling between the two spins, with the dipolar coupling having an inverse cubed dependence on the internuclear distance: the presence or absence of DQ correlation peaks is indicative of the close proximity, typically up to 3.5 Å, or not of two hydrogen atoms.^98,106,107^

Consider the two highest ppm ^1^H resonances at 12.8 and 13.6 ppm corresponding to the H7 and H1 NH, for which strong ^14^N–^1^H correlation peaks were observed in Fig. 1. For the H7 SQ ^1^H resonance, there is one pair of DQ peaks at 12.8 + 8.4 = 21.2 ppm, while for the H1 SQ ^1^H resonance, there are two pairs of DQ peaks at 13.6 + 9.2 = 22.8 ppm and at 13.6 + 4.2 = 17.8 ppm. On the basis of the GIPAW calculation of ^1^H chemical shifts for the geometry optimised crystal structure of 1, these are assigned to intramolecular H–H proximities (see Table 3) of the NH H7 to the CH H8 neighbour in the same aromatic ring (at 8.4 ppm) and between the NH H1 and the CH H2 neighbour in the same aromatic ring (at 9.2 ppm) and between the NH H1 and the CH H11 of the adjacent ring (at 4.2 ppm).

**Table 3:** H–H proximities^a^ (<3.5 Å) in 1 corresponding to experimentally observed ^1^H DQ frequencies as seen in Fig. 3

Proton 1	*δ*_SQ1_ (ppm)	Proton 2	*δ*_SQ2_ (ppm)	*δ*_DQ_ (ppm)	Separation (Å)
13b (CH_3_)	0.2	14 (CH)	3.3	3.5	2.49
17 (CH_3_)	0.6	14 (CH)	3.3	3.9	2.47, 2.47, 3.07
17 (CH_3_)	0.6	16b (CH_2_)	3.5	4.1	2.42, 3.31
12b (CH_2_)	0.7	16b (CH_2_)	3.5	4.2	2.65
13a (CH_2_)	1.0	14 (CH)	3.3	4.3	2.36
17 (CH_3_)	0.6	21b (CH_2_)	3.8	4.4	2.47, 2.70
32 (CH_3_)	2.4	32 (CH_3_)	2.4	4.8	1.78, 1.78
12a (CH_2_)	0.7	11 (CH)	4.3	5.0	2.50
12b (CH_2_)	0.7	11 (CH)	4.3	5.0	3.07
13a (CH_2_)	1.0	11 (CH)	4.3	5.3	2.49
32 (CH_3_)	2.4	11 (CH)	4.3	6.7	2.97
16b (CH_2_)	3.5	11 (CH)	4.3	7.8	3.05
14 (CH)	3.3	20 (CH)	4.4	7.7	1.88
32 (CH_3_)	2.4	16a (CH_2_)	5.3	7.7	3.21, 2.80, 2.64
32 (CH_3_)	2.4	21a (CH_2_)	5.4	7.8	3.16
21b (CH_2_)	3.8	20 (CH)	4.4	8.2	2.43
17 (CH_3_)	0.6	26 (CH)	7.6	8.2	2.77
32 (CH_3_)	2.4	25 (CH)	6.2	8.6	2.53, 3.00
12a (CH_2_)	0.7	22 (CH)	7.8	8.5	3.09
13a (CH_2_)	1.0	22 (CH)	7.8	8.8	2.93
16b (CH_2_)	3.5	16a (CH_2_)	5.3	8.8	1.77
21b (CH_2_)	3.8	21a (CH_2_)	5.4	9.2	1.87
11 (CH)	4.3	16a (CH_2_)	5.3	9.6	2.43
32 (CH_3_)	2.4	23 (CH)	7.5	9.9	2.45, 3.31
32 (CH_3_)	2.4	8 (CH)	8.4	10.8	2.90, 2.69
32 (CH_3_)	2.4	9 (CH)	8.5	10.9	3.02, 2.96
26 (CH)	7.6	16b (CH_2_)	3.5	11.1	2.70
32 (CH_3_)	2.4	2 (CH)	9.2	11.6	2.63, 3.17
32 (CH_3_)	2.4	10 (NH)	9.2	11.6	3.20, 3.34
16b (CH_2_)	3.5	8 (CH)	8.4	11.9	2.63
16b (CH_2_)	3.5	10 (NH)	9.2	12.7	2.31
11 (CH)	4.3	10 (NH)	9.2	13.5	2.95
16a (CH_2_)	5.3	8 (CH)	8.4	13.7	2.17
25 (CH)	6.2	26 (CH)	7.6	13.8	2.48
21a (CH_2_)	5.4	8 (CH)	8.4	13.8	3.43
25 (CH)	6.2	22 (CH)	7.8	14.0	2.71
16a (CH_2_)	5.3	2 (CH)	9.2	14.5	2.90
16a (CH_2_)	5.3	10 (NH)	9.2	14.5	2.71
23 (CH)	7.5	22 (CH)	7.8	15.3	2.49
26 (CH)	7.6	22 (CH)	7.8	15.4	2.71
8 (CH)	8.4	9 (CH)	8.5	16.9	2.70
9 (CH)	8.5	10 (NH)	9.2	17.7	2.56
9 (CH)	8.5	2 (CH)	9.2	17.7	3.18
11 (CH)	4.3	1 (NH)	13.6	17.9	2.13
8 (CH)	8.4	7 (NH)	12.8	21.2	2.51
2 (CH)	9.2	1 (NH)	13.6	22.8	2.25

a The proximities were extracted from the DFT geometry-optimised (CASTEP) crystal structure of 1 (CCDC 2352028). Note that intra-CH_3_ and intra-CH_2_ H–H proximities for atoms 12, 13 and 17 that correspond to the broad peak at ∼1 + ∼1 = ∼2 ppm are not listed.

**Fig. 4:**
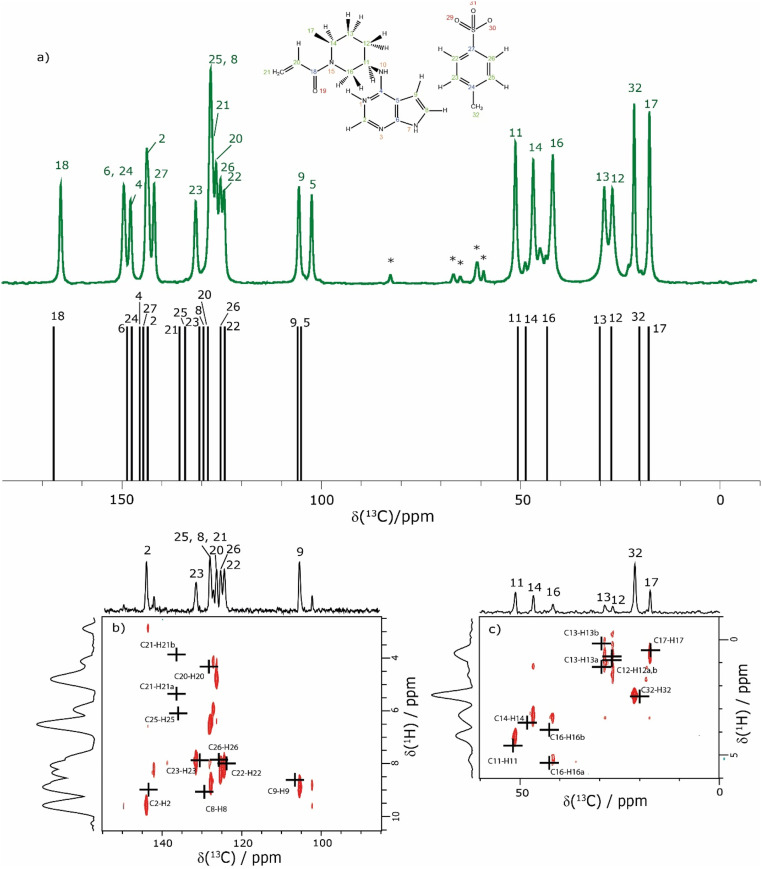
(a) A 1D ^1^H (600 MHz)–^13^C CP (2 ms) MAS (12.5 kHz) NMR spectrum (top) of 1 acquired with 2048 co-added transients. The asterisks denote spinning sidebands. The stick spectrum (bottom) represents the GIPAW calculated ^13^C chemical shifts for the DFT (CASTEP) geometry-optimised structure of 1 (CCDC 2352028, see Table 4). (b) and (c) Two-dimensional ^1^H (1 GHz)–^13^C CP (500 μs) HETCOR MAS (60 kHz) NMR spectra with skyline projections for the aromatic and aliphatic regions, respectively. Here, the low-power ^13^C irradiation during CP was at an irradiation frequency of (b) 120 ppm and (c) 50 ppm. The black crosses in (b) and (c) represent the GIPAW calculated chemical shifts for the directly bonded CH connectivities up to 1.1 Å. The base contour level is at 17% and 14% of the maximum peak height for (b) and (c), respectively.

The assignment of the CH ^1^H resonances is aided by the two-dimensional ^1^H–^13^C heteronuclear correlation (HETCOR) solid-state NMR spectra of 1 presented in Fig. 4b and c. These spectra were recorded using a pulse sequence whereby CP was employed to transfer magnetisation from ^1^H to ^13^C *via*^13^C–^1^H heteronuclear dipolar couplings. Note that, for this experimental implementation at 60 kHz MAS and a ^1^H Larmor frequency of 1 GHz, a low ^13^C nutation frequency of 10 kHz was applied during CP such that the presented spectra had to be separately recorded for the high-ppm (aromatic) and low-ppm (aliphatic) regions, as presented in Fig. 4b and c, respectively. Fig. 4 additionally presents in Fig. 4a a one-dimensional ^1^H (600 MHz)–^13^C CP MAS (12.5 kHz) NMR spectrum of 1 that was recorded with a CP contact time of 2 ms. Asterisks in Fig. 4a denote spinning sidebands that are observed at 83 ppm (corresponding to 12.5 kHz at the ^13^C Larmor frequency of 150.9 MHz) away from the centreband for carbonyl, aromatic and alkene ^13^C resonances that exhibit large chemical shift anisotropies.

The CP contact time was 2 ms for the one-dimensional ^1^H–^13^C CP MAS (12.5 kHz) NMR spectrum in Fig. 4a and 500 μs for the 2D ^1^H–^13^C CP-HETCOR MAS NMR spectra in Fig. 4b and c. As discussed above for the ^1^H–^15^N CP MAS NMR spectrum in Fig. 1b, solid-state NMR spectra recorded using CP are not quantitative in that the peak intensities in the ^1^H–^13^C CP MAS NMR spectrum depend on the transfer of transverse magnetisation from ^1^H to ^13^C during the CP contact time. For the CP contact time of 500 μs as used to record the CP-HETCOR MAS NMR spectra, resonances are predominantly observed in Fig. 4b and c for the protonated CH, CH_2_ and CH_3_ resonances. By comparison, for the CP contact time of 2 ms as used to record the one-dimensional CP MAS NMR spectrum in Fig. 4a, similar intensity is observed for the protonated and non-protonated resonances.

In Fig. 4, the results of the GIPAW calculation for the DFT (CASTEP) geometry-optimised crystal structure of 1 are represented by a stick spectrum in Fig. 4a for the calculated ^13^C chemical shifts and by black crosses in Fig. 4b and c for the calculated ^1^H and ^13^C chemical shifts for the CH, CH_2_ and CH_3_ moieties. Table 4 lists the assigned experimental and GIPAW calculated ^1^H and ^13^C chemical shifts for 1. For the aliphatic resonances, *i.e.*, those with a ^13^C chemical shift below 55 ppm, there is good agreement between solid-state NMR experiment and GIPAW calculation: for ^13^C, the biggest discrepancy compared to experiment is for C14 at 2.0 ppm, while for ^1^H, the biggest discrepancy is 0.4 ppm for H16b (see Fig. 4a and c and Table 4). For the high ppm (>100 ppm) ^13^C resonances, the ^1^H–^13^C CP-HETCOR MAS NMR spectrum in Fig. 4b enables the distinguishing of protonated and non-protonated carbon atoms for which the ^13^C chemical shifts are similar, namely the C9 CH at 105.6 ppm from the C5 C at 102.3 ppm, as well as the C2 CH at 143.6 ppm from the C27 C at 141.8 ppm. Specifically, high intensity C9–H9 and C2–H2 cross peaks are observed for the directly bonded pairs of ^13^C and ^1^H at (105.6 ppm, 8.5 ppm) and (143.6 ppm, 9.2 ppm), respectively. By comparison, only weak intensity cross peaks are observed for proximities between the non-protonated C5 C at 102.3 ppm with H9 (at 8.5 ppm) that is attached to the neighbouring C9 atom of the 5-membered ring, and between the non-protonated tosylate C27 C at 141.8 ppm with H22 (at 7.8 ppm) and H26 (at 7.6 ppm) that are attached to the neighbouring C22 and C26 atoms of the phenyl ring.

**Table 4:** Experimental solid-state and GIPAW calculated ^1^H and ^13^C NMR chemical shifts (in ppm) for 1

Atom no.	Solution-state^a^		Solid-state		GIPAW calculated^b^	
	^1^H	^13^C	^1^H	^13^C	^1^H	^13^C
1 (NH^+^)	13.44	—	13.6	—	14.3	—
2 (CH)	8.39	142.7	9.2	143.6	9.0	143.6
4 (C)	—	149.9	—	147.9	—	145.8
5 (C)	—	101.7	—	102.3	—	105.6
6 (C)	—	145.0	—	149.5	—	148.4
7 (NH)	12.67	—	12.8	—	14.7	—
8 (CH)	7.44	124.4	8.4	127.9	9.1	129.7
9 (CH)	6.93	101.5	8.5	105.6	8.6	106.6
10 (NH)	9.19	—	9.2	—	9.9	—
11 (CH)	3.97 (4.00)	48.2 (48.9)	4.3	51.0	4.6	51.1
12 (CH_2_)	1.96–1.80	24.7	0.7	26.6	0.9, 0.7	27.4
13 (CH_2_)	1.80–1.61	28.8 (27.8)	1.0, 0.2	28.7	1.2, 0.2	29.8
14 (CH)	4.41 (4.81)	46.6 (42.3)	3.3	46.5	3.6	48.5
16 (CH_2_)	4.54, 2.80 (4.11, 3.14)	39.3 (43.3)	5.3, 3.5	41.6	5.3, 3.9	42.9
17 (CH_3_)	1.23 (1.16)	16.4 (14.9)	0.6	17.3	0.5^c^	17.4
18 (C=O)	—	165.0 (164.5)	—	165.4	—	166.5
20 (CH)	6.85	128.9 (128.7)	4.4	126.1	4.3	128.0
21 (CH_2_)	6.12, 5.72 (6.12, 5.87)	127.2 (127.4)	5.4, 3.8	127.5	5.4, 3.8	136.5
22 (CH)	7.49	125.4	7.8	124.3	8.0	124.1
23 (CH)	7.12	128.0	7.5	131.6	7.8	131.1
24 (C)	—	145.4	—	149.6	—	147.8
25 (CH)	7.12	128.0	6.2	127.9	6.1	135.8
26 (CH)	7.49	125.4	7.6	125.3	7.8	125.6
27 (C)	—	137.6	—	141.8	—	144.8
32 (CH_3_)	2.29	20.7	2.4	21.3	2.5^c^	20.0

a Solution-state data was measured in DMSO. (Brackets indicate chemical shifts for the *trans* rotamer around the amine bond.)

b GIPAW calculated values for the geometry-optimised crystal structure of 1 (CCDC 2352028). A reference shielding value of 172.0 ppm was used for all ^13^C atoms above 45 ppm, whilst for the ^13^C atoms below 45 ppm, a reference shielding value of 175.0 ppm was used.^91^ In the case of ^1^H, a reference value of 31 ppm was used.

c In the case of the methyl groups, an average value is reported for the ^1^H GIPAW calculated chemical shifts.

The most crowded part of the ^1^H–^13^C CP-HETCOR MAS NMR spectrum in Fig. 4b is between ^13^C chemical shifts of 120 and 140 ppm corresponding to aromatic CH and alkene CH and CH_2_ resonances. Moreover, this is where the greatest discrepancy between experiment and GIPAW calculation is observed. Considering ^1^H chemical shifts below 6.5 ppm, four cross peaks are expected for the C25–H25 tosylate pair and the C20–H20, C21–H21a and C21–H21b alkene pairs. In Fig. 4b, experimental cross peaks are observed for ^13^C chemical shifts between 126.1 ppm and 127.9 ppm for ^1^H chemical shifts below 6.5 ppm, while the GIPAW calculated ^13^C chemical shifts are 128.0, 136.5 and 135.8 ppm for C20, C21 and C25, respectively. For the assignment in Table 4, there is a discrepancy of 9.0 and 7.9 ppm for C21 and C25. The biggest discrepancy for ^1^H is for the C8 CH, where the experimental and GIPAW calculated ^1^H chemical shifts are 8.4 and 9.1 ppm, respectively.

Table 4 lists both solution (DMSO) and solid-state NMR chemical shifts for 1. The differences between experimental solution- and solid-state NMR ^13^C chemical shifts are mostly within ±2 ppm, as was the case for the discrepancy between most experimental solid-state and GIPAW calculated ^13^C chemical shifts discussed above. The biggest difference between solid-state and solution ^13^C chemical shifts is 4.5 ppm for C6. Greater variation as compared to the much smaller range of chemical shifts (∼20 ppm for ^1^H compared to ∼200 ppm for ^13^C) is observed for the ^1^H chemical shifts, noting the greater sensitivity of the ^1^H chemical shift to the solid-state packing, *e.g.*, ring currents from the aromatic groups. Variations of more than 1 ppm are observed for the H9 CH and the H20 CH with solution and solid-state ^1^H chemical shifts of 6.93 ppm and 8.5 ppm for H9 and 6.85 ppm and 4.4 ppm for H20.

**Fig. 5:**
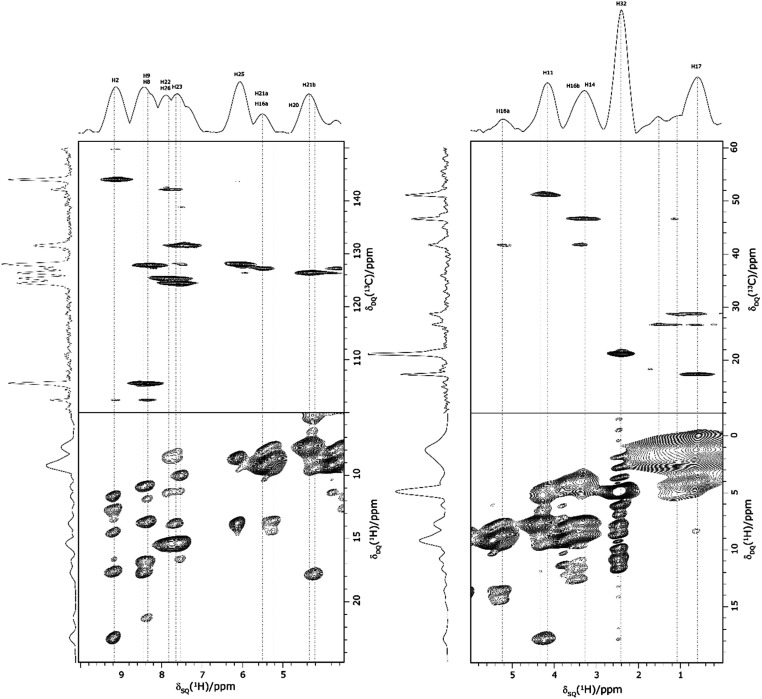
2D MAS (60 kHz) NMR spectra with skyline projections of 1 recorded at 1 GHz. Top: ^1^H–^13^C CP HETCOR spectra for the high (left) and low (right) ppm regions repeated from Fig. 4b and c, respectively. Bottom: Corresponding regions of the ^1^H–^1^H DQ–SQ spectrum repeated from Fig. 3. Note that the ^1^H–^13^C CP HETCOR spectra have been rotated through 90° so as to achieve the alignment of the ^1^H SQ axis as horizontal for both sets of spectra.

Returning to the ^1^H–^1^H DQ–SQ MAS NMR spectrum of 1 that was presented in Fig. 3, it is evident that the assignment of the ^1^H SQ resonances in Fig. 3 follows from the assignment of the CH correlation peaks in the ^1^H–^13^C CP-HETCOR MAS NMR spectra that were presented in Fig. 4b and c. This is further shown in Fig. 5 that presents the ^1^H–^13^C CP-HETCOR MAS NMR spectra (top) with the ^1^H–^1^H DQ–SQ MAS NMR spectra (bottom), whereby the HETCOR spectra have been rotated through 90° such that there is a common horizontal ^1^H SQ chemical axis.

## Case study 2: cellulose polymorphs

In ref. 5, Simmons *et al.* employed GIPAW calculation of ^13^C NMR chemical shifts for 10 residue DFT-optimised molecular dynamics generated xylan structures to confirm that changes observed experimentally for the ^13^C NMR chemical shifts for xylan are sensitive to the adoption of a two- and three-fold screw. As shown in Table 1 of ref. 5, agreement between experiment and GIPAW calculation for the change in ^13^C NMR chemical shift varied from within 0.8 ppm to within 3.4 ppm. In the context also of the observation of six different glucose environments with distinct ^13^C NMR chemical shifts that are common to the cellulose in a range of plants (poplar wood, spruce wood and grasses), it is interesting to consider, in Table 5, the discrepancy between experiment and GIPAW calculation for the two cellulose polymorphs for which crystal structures are available, cellulose Iα and cellulose Iβ.^108,109^ Note that in both cases there are two distinct molecules in the asymmetric unit cell. Specifically, in cellulose Iα, the two distinct molecules are neighbouring molecules within the same chain, whereas, in cellulose Iβ, the two distinct molecules correspond to separate chains, called centre and origin. Table 5 presents calculated ^13^C NMR chemical shifts for three different calculation approaches and compares the GIPAW calculations to experimental ^13^C NMR chemical shifts reported by Brouwer and Mikolajewski.^110,111^ Two calculations using the PBE functional are presented: one with an incompletely converged geometry optimisation (as in the submitted article made available ahead of the discussion meeting) and one with converged geometries. In addition, results are presented for a calculation using the meta-GGA rSCAN for both the geometry optimisation and the GIPAW NMR calculation. It is evident that better agreement to experiment is observed when using rSCAN with both reduced RMSD and maximum difference. We note that GIPAW calculations of mono- and disaccharides have also been reported by Yates *et al.* for maltose,^29^ by Brouwer *et al.* for glucose^112^ and by Kibalchenko *et al.* for galactose,^113^ while quantum-chemical calculations have been presented for fragments of the cellulose structures by Wang *et al.*^114^

**Table 5:** Comparison of GIPAW calculated ^13^C NMR chemical shifts (in ppm) for cellulose polymorphs to experiment

	Cellulose Iα				Cellulose Iβ			
	GIPAW^a^			Expt^b^	GIPAW^a^			Expt^b^
	PBE (not conv.)	PBE (conv.)	rSCAN		PBE (not conv.)	PBE (conv.)	rSCAN	
**Unit 1**	**C7–C12**				**C1, C3,** …			
C1	107.6	108.2	105.4	105.6	106.6	108.2	105.6	104.4
C2	74.0	74.4	74.8	72.2	73.3	72.8	73.5	71.7
C3	74.4	74.8	75.5	74.6	73.2	72.0	73.4	75.3
C4	87.3	86.1	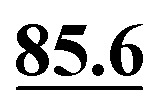	89.4	87.9	90.2	89.4	88.4
C5	70.8	71.8	72.7	73.1	71.0	69.4	70.6	71.4
C6	65.3	65.0	65.5	65.7	64.4	64.0	64.9	66.0

**Unit 2**	**C1–C6**				**C13, C15,** …			
C1	107.6	106.9	104.2	105.5	108.7	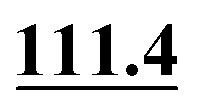	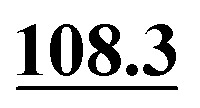	106.1
C2	75.4	74.1	74.4	71.2	70.7	70.5	71.4	71.7
C3	75.1	74.0	74.5	75.1	75.3	74.1	75.1	74.4
C4	93.6	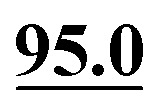	93.7	90.3	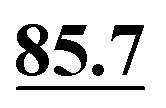	88.1	87.4	89.2
C5	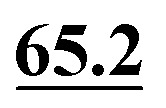	66.9	68.0	71.3	74.5	71.2	72.2	72.9
C6	63.3	64.0	65.0	65.8	65.3	64.5	65.2	65.2
*σ*_*r*ef_ c	168.1	168.1	178.5		168.1	168.7	178.0	
RMSD	2.8	2.6	2.2		1.8	2.5	1.3	
Max. diff.^d^	6.1	4.7	3.8		3.5	5.3	2.1	

a The crystal structures for cellulose Iβ (JINROO05, 792796)^108^ and cellulose Iβ (JINROO01, 810 597)^109^ were used as starting points for geometry optimisation.

b Experimental values are taken from Brouwer and Mikolajewski.^110,111^ with C1 chemical shifts switched for cellulose Iβ^115^ Assignment to unit 1 and unit 2 is based on the relative change in the C1 and C5 ^13^C chemical shift

c *δ*_calc._ = *σ*_ref_ − *σ*_calc_.

d The ^13^C chemical shift with the maximum discrepancy between experiment and GIPAW calculation is indicated in underlined bold.

## Summary and outlook

This article has presented two case studies of the application of NMR crystallography of organic molecules to two important research areas, namely pharmaceuticals and plant cell walls. Building upon 20 years of literature applications, these two case studies showcase the great value of DFT calculation using the GIPAW method, in complementing experimental solid-state NMR. While agreement with experiment is good, indeed remarkably good given the inherent approximations of DFT, the discrepancy that typically corresponds to 1% of the chemical shift range for ^1^H and ^13^C is nevertheless restrictive, for example in seeking to provide evidence for different structural models for plant cell walls where there are only subtle changes in chemical shift. There is thus much motivation for continued innovation in the field of NMR crystallography.

## Data availability

The calculated and experimental data for this study are provided as a supporting data set from WRAP, the Warwick Research Archive Portal at http://wrap.warwick.ac.uk/188293.

## Conflicts of interest

Some of the authors are employees of Pfizer, and compound 1 is a Pfizer pharmaceutical.

## Supplementary Material

FD-255-D4FD00088A-s001

FD-255-D4FD00088A-s002
