# Supplementary material for: Organic NMR crystallography: enabling progress for applications to pharmaceuticals and plant cell walls
Source: Faraday Discuss. 2024 May 20;255:222–43. doi: 10.1039/d4fd00088a (PMC11599944; doi:10.1039/d4fd00088a)
Supplement: FD-255-D4FD00088A-s001 [file FD-255-D4FD00088A-s001.pdf]

Supplementary Information (SI) for Faraday Discussions.
This journal is © The Royal Society of Chemistry 2024

Organic NMR Crystallography: Enabling Progress for Applications to Pharmaceuticals and Plant Cell Walls: Supporting Information

Zainab Rehman,[a] Jairah Lubay,[a] W. Trent Franks,[a] Emily K. Corlett,[b] Albert P. Bartók,[c,d] Bao Nguyen,[c] Garry Scrivens,[b] Brian M. Samas,[c] Heather Frericks-Schmidt,[c] and Steven P. Brown[a]*

a Department of Physics, University of Warwick, Coventry, CV4 7AL, UK.
b Pfizer Worldwide R&D, Sandwich, Kent, UK.
c Pfizer Worldwide R&D, Groton, CT, USA.
d Warwick Centre for Predictive Modelling, School of Engineering, University of Warwick, Coventry, CV4 7AL, UK
*E-mail: S.P.Brown@warwick.ac.uk

S1. Single Crystal X-ray Diffraction

The single crystal X-ray structure of Ritlectinib Tosylate was determined at 296 K in the monoclinic system, space group P21 (see Table S1). Data collection was performed on an Bruker APEX II CCD using a CuKα X-ray source at 1.54178 Å. 15363 reflections were collected using omega and phi scans. The structure was solved using the SHELXTL program and a full-matrix least-squares refinement on F^2. All non-hydrogen atoms were refined anisotropically. All carbon-bound hydrogens were placed in geometrically calculated positions and refined using a riding model. Hydrogen atoms on heteroatoms were refined with isotropic displacement parameters. The final R-index is 2.17%. The absolute stereochemistry was determined with a Flack parameter of 0.05(4). Structural diagrams in this paper were created using Mercury 4.2.0.[1] Figure S1 shows the packing of the molecule along the *a* axis. The blue and red molecules represent the free base and the tosylate salt, respectively.

Table S1. Selected single crystal X-ray diffraction data for the geometry-optimised crystal structure of **1** (CCDC 2352028)

	Ritlectinib Tosylate
Stoichiometry (base : acid)	1 : 1
Chemical Formula	$C_{22}H_{27}N_5O_4S$
Formula Weight (g mol^{-1})	457.55
Crystal System	Monoclinic

Space Group	$P2_1$
a (Å)	9.1890(4)
b (Å)	12.6757(6)
c (Å)	10.9694(5)
α *(°)*	90.0000
β *(°)*	114.0130(2)
γ *(°)*	90.0000
Z	2
Temperature (K)	296(2)
R1 [I > 2σ(I)]	0.0217

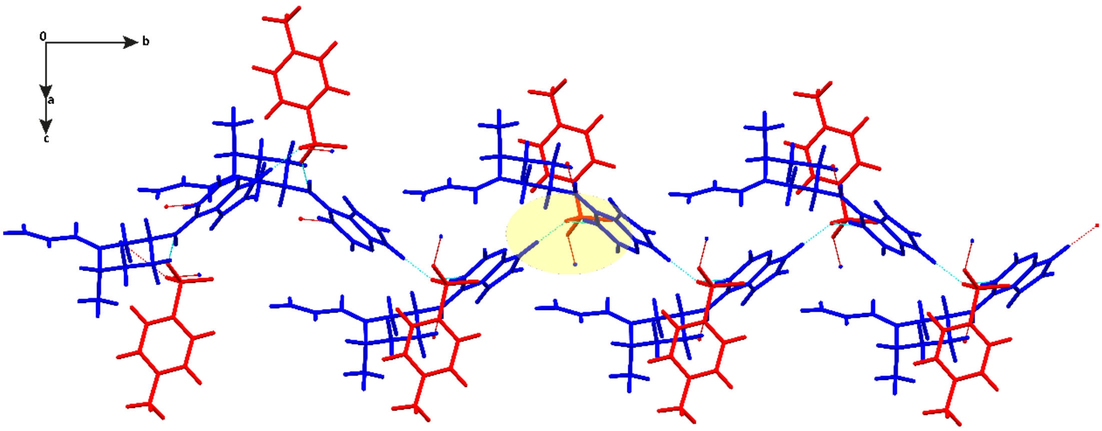

Figure S1. Packing of **1** along the *a* axis for the geometry-optimised crystal structure of **1** (CCDC 2352028). The blue and red molecules represent the free base and the tosylate salt, respectively. The NH…O hydrogen bonding interactions are represented by cyan dashed lines. The highlighted yellow section is magnified and shown in Figure 2 in the main text.

S2. Powder X-Ray Diffraction

PXRD was performed on a Panalytical X-PertPro MPD with a curved Ge Johansson monochromator, giving pure Cu Kα1 (1.5406 Å) radiation and a solid-state PiXcel detector. Each sample was loaded onto a zero-background Si-holder rotating at 30 rpm. Each step size was 0.013° resulting in a total experiment time of 60 minutes.

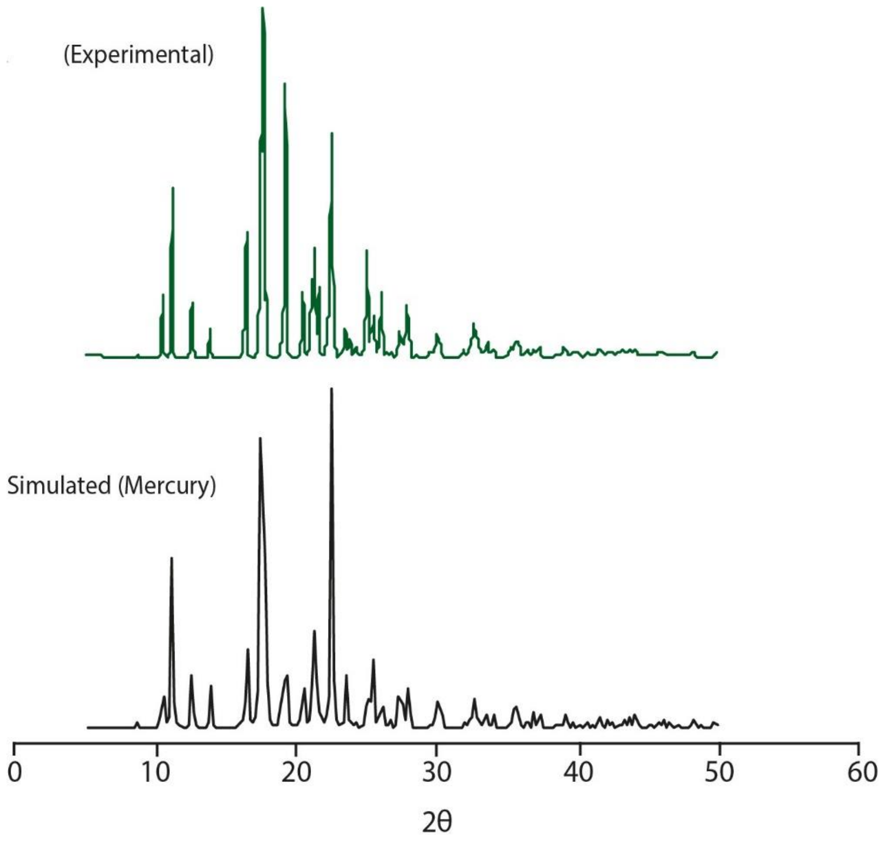

Figure S2. Experimental PXRD data for **1** (green) compared to the predicted values (black) generated using Mercury from the.cif file for the geometry-optimised crystal structure of **1** (CCDC 2352028).

S3. Additional Solid-State NMR Spectra of 1

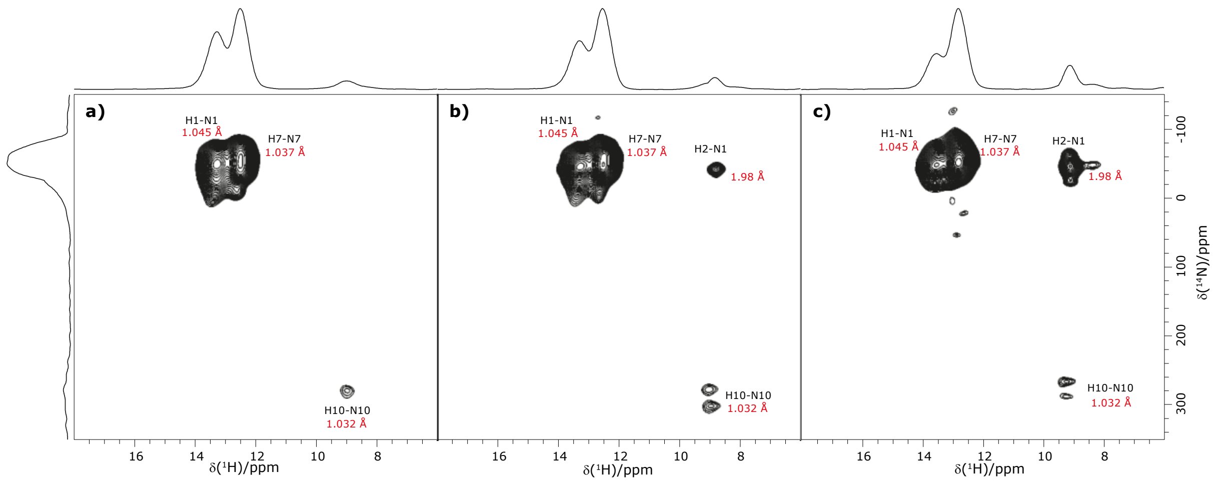

Figure S3. ^{14}N-^{1}H(600 MHz) HMQC MAS NMR spectra with skyline projections of **1** recorded with 8, 16 and 24 rotor periods of R^3 recoupling. τ_{RCPL} = 133.7, 267.2 and 400.8 μs.

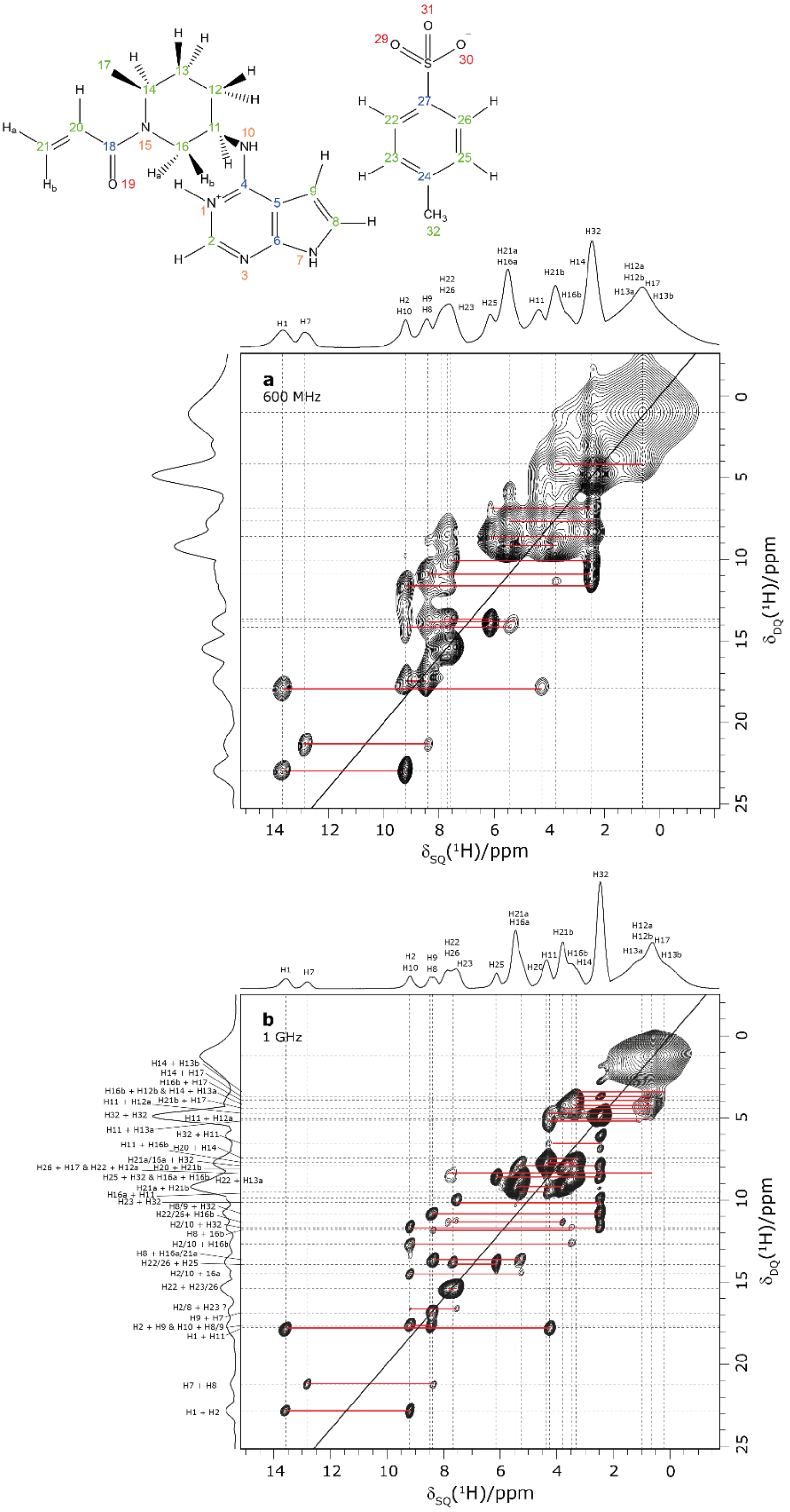

Figure S4. ^{1}H DQ-SQ 2D MAS (60 kHz) NMR spectra of **1** with skyline projections recorded with one rotor period of BaBa recoupling at a [1]H Larmor frequency of (a) 600 MHz and (b) 1 GHz. The base contour levels are at (a) 8% and b) 4% of the maximum peak height, respectively.

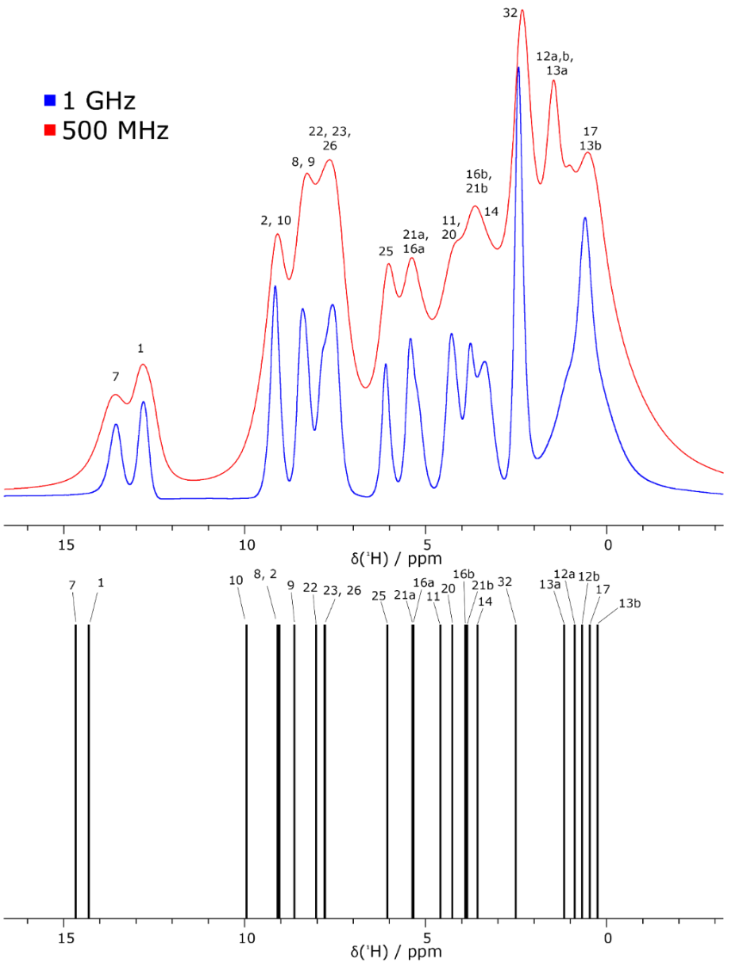

Figure S5. (Top) One-pulse ^{1}H MAS (60 kHz) NMR spectra of **1** recorded at a ^{1}H Larmor frequency of 500 MHz (red) and 1 GHz (blue). 32 and 16 transients were co-added at 500 MHz and 1 GHz, respectively. (Bottom) A stick spectrum representing the GIPAW calculated chemical shifts (see Table 4 in the main text). Assignments are based on the two-dimensional ^{1}H-^{13}C, ^{14}N-^{1}H and ^{1}H-^{1}H DQ-SQ correlation spectra presented in Figures 4, 1 and 3 of the main text, respectively.

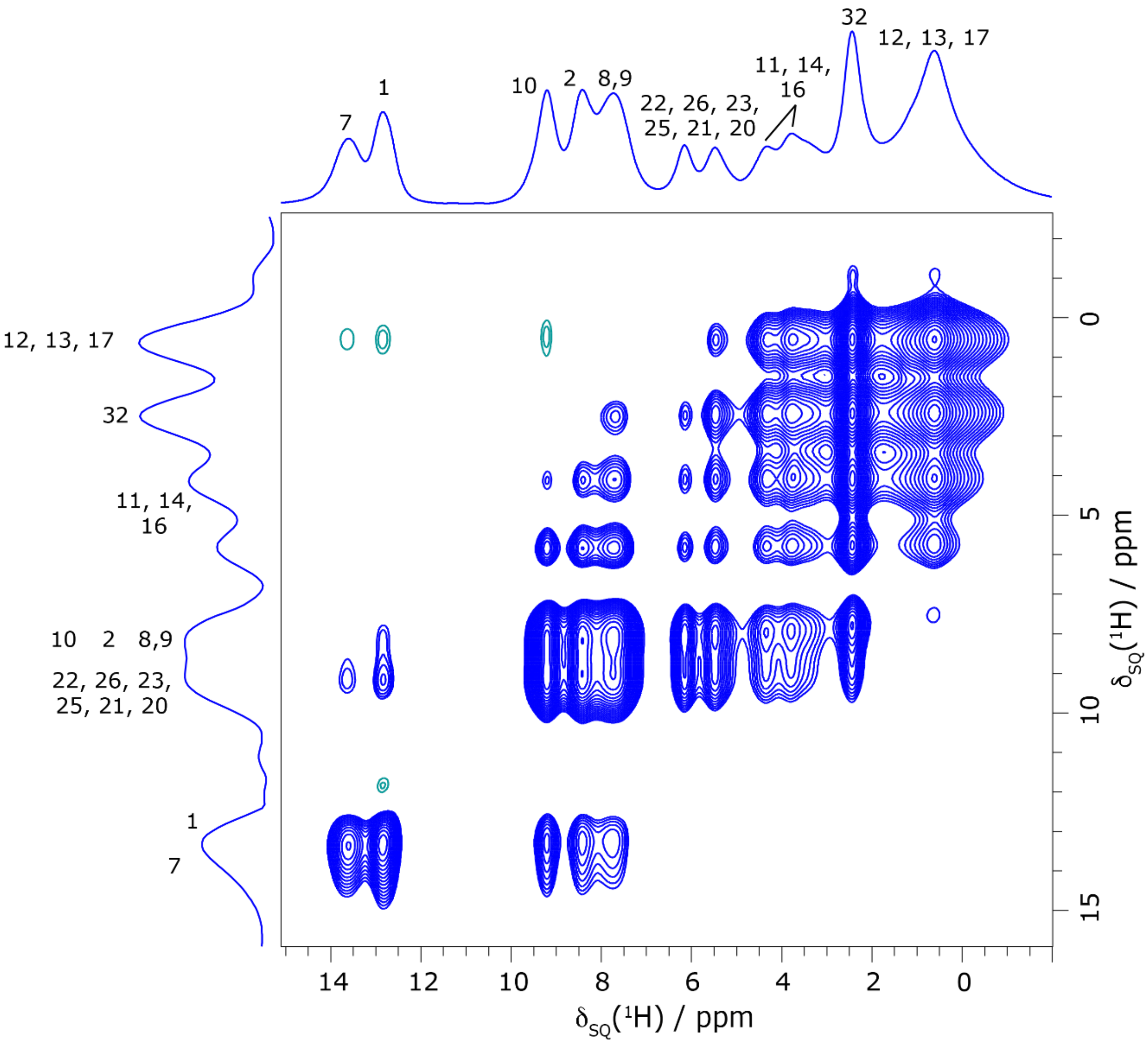

Figure S6. A ^{1}H(600 MHz)-^{1}H SQ-SQ 2D MAS (60 kHz) NMR spectrum for **1**. This was recorded using a NOESY-like spin diffusion pulse sequence with t_{mix} = 50 ms.

1. C. F. Macrae, P. R. Edgington, P. McCabe, E. Pidcock, G. P. Shields, R. Taylor, M. Towler and J. van De Streek, *J. Appl. Crystallogr.*, 2006, **39**, 453.
